# Antioxidant and Anti-Inflammatory Effects of Blueberry Anthocyanins on High Glucose-Induced Human Retinal Capillary Endothelial Cells

**DOI:** 10.1155/2018/1862462

**Published:** 2018-02-22

**Authors:** Wuyang Huang, Zheng Yan, Dajing Li, Yanhong Ma, Jianzhong Zhou, Zhongquan Sui

**Affiliations:** ^1^Institute of Farm Product Processing, Jiangsu Academy of Agricultural Sciences, Nanjing 210014, China; ^2^Jiangsu Key Laboratory for Horticultural Crop Genetic Improvement, Jiangsu Academy of Agricultural Sciences, Nanjing 210014, China; ^3^Department of Food Science and Engineering, School of Agriculture and Biology, Shanghai Jiao Tong University, Shanghai 200240, China

## Abstract

Blueberries possess abundant anthocyanins, which benefit eye health. The purpose of this study was to explore the protective functional role of blueberry anthocyanin extract (BAE) and its predominant constituents, malvidin (Mv), malvidin-3-glucoside (Mv-3-glc), and malvidin-3-galactoside (Mv-3-gal), on high glucose- (HG-) induced injury in human retinal capillary endothelial cells (HRCECs). The results showed that BAE, Mv, Mv-3-glc, and Mv-3-gal enhanced cell viability (*P* < 0.05 versus the HG group at 24 h); decreased the reactive oxygen species (ROS, *P* < 0.01 versus the HG group both at 24 and 48 h); and increased the enzyme activity of catalase (CAT) and superoxide dismutase (SOD) (*P* < 0.05 versus the HG group both at 24 and 48 h). Mv could greatly inhibit HG-induced Nox4 expression both at 24 and 48 h (*P* < 0.05), while BAE and Mv-3-gal downregulated Nox4 only at 48 h (*P* < 0.05). Mv, Mv-3-glc, and Mv-3-gal also changed nitric oxide (NO) levels (*P* < 0.05). BAE and Mv-3-glc also influenced angiogenesis by decreasing the vascular endothelial cell growth factor (VEGF) level and inhibiting Akt pathway (*P* < 0.05). Moreover, Mv and Mv-3-glc inhibited HG-induced intercellular adhesion molecule-1 (ICAM-1, *P* < 0.001) and nuclear factor-kappa B (NF-*κ*B) (*P* < 0.05). It indicated that blueberry anthocyanins protected HRCECs via antioxidant and anti-inflammatory mechanisms, which could be promising molecules for the development of nutraceuticals to prevent diabetic retinopathy.

## 1. Introduction

Diabetic retinopathy (DR) is a common complication of diabetes mellitus and a leading cause of vision loss and blindness in diabetic patients [1]. Hyperglycemia is known to be the main contributing factor in the progression of the disease, which triggers pathological metabolic and biochemical changes that damage the retinal cells [2]. In the development of DR, retinal microvascular dysfunction includes the loss of endothelial cells and pericytes, capillary occlusion and blood-retinal barrier breakdown, and endothelial cell hypertrophy and degeneration that lead to capillary nonperfusion and hypoxia [3, 4].

Dietary intake of phytochemicals, particularly anthocyanins, is being increasingly recognized as beneficial for modern human eye health [5]. Anthocyanins are known to have antioxidant, antimicrobial, antiviral, antiallergic, anticarcinogenic, anti-inflammatory, antimutagenic, and antiproliferative effects and thus may play an essential role in preventing various degenerative diseases including DR [6]. Anthocyanins from different vegetables and fruits, such as blueberry, bilberry, mulberry, maqui berry, blackcurrant, and black soybean, could reduce retinal degeneration and improve visual function [7–10]. Their possible functional mechanisms include the attenuation of oxidative damage, alteration of retinal enzyme activity, inhibition of inflammation, stimulation of the immune system, reduction of platelet aggregation, and modulation of cholesterol synthesis and hormone metabolism [11, 12]. More and more scientists and consumers realize the vision-related benefits of anthocyanin-rich berries, and the anthocyanosides from berries are currently used in ophthalmology for their capacity to improve vision and prevent diabetic retinopathy [13].

Blueberries are rich in anthocyanins, whose content is believed to be the highest among all commonly consumed vegetables and fruits [14]. Blueberries are famous for their wide range of health benefits, including ameliorating diabetes, attenuating vascular problems, maintaining endothelial function, and preventing retinal diseases [15]. Many scientists have reported that blueberry components such as anthocyanins, polyphenols, and pterostilbene can protect corneal epithelial cells against light-induced retinal injury via antioxidative, antiangiogenic, and antiaging effects in vitro, and the bioactivity of blueberries in improving vision has been confirmed in vivo [13, 16, 17]. However, the function of blueberry anthocyanins on human retinal capillary endothelial cells (HRCECs) is still unknown. Liu et al. identified anthocyanin components of wild Chinese blueberries and found that malvidin glycosylated with hexose or pentose accounted for >46% of the total anthocyanin content in blueberries [18]. Our previous survey also confirmed that malvidin-3-glucoside (Mv-3-glc) and malvidin-3-galactoside (Mv-3-gal) were major anthocyanins in Brightwell rabbiteye blueberry (*Vaccinium ashei*) of Nanjing [19]. In the present study, the protective effects of blueberry anthocyanin extract, as well as malvidin and its glycosides (Mv-3-glc and Mv-3-gal) on high glucose-induced injury in HRCECs, were investigated to propose a functional mechanism for the role of antioxidants in eye nourishment.

## 2. Materials and Methods

### 2.1. Materials and Chemicals

Brightwell rabbiteye blueberry (*Vaccinium ashei*) was harvested in July 2016 from the orchards of Lishui in Nanjing. The plant species were authenticated by a blueberry expert at the Institute of Botany, Jiangsu Province and the Chinese Academy of Sciences. The collected fruits were kept at −18°C in the dark.

Human retinal capillary endothelial cells (HRCECs, primary culture cells) were purchased from Jennio Biotechnology Co. Ltd. (Guangzhou, China). Malvidin (Mv), malvidin-3-glucoside (Mv-3-glc), malvidin-3-galactoside (Mv-3-gal), and trypsin were purchased from Sigma Aldrich (Shanghai, China). Fetal bovine serum (FBS) and DMEM medium were obtained from Gibco/Invitrogen (Shanghai, China). Penicillin and streptomycin were purchased from Life Technologies (Shanghai, China). Reactive oxygen species (ROS) Assay Kit, BCA (Bicinchoninic acid) Protein Assay Kit, and MTT Cell Proliferation Kit were purchased from Beyotime Institute of Biotechnology (Shanghai, China). Andygene human catalase (CAT), superoxide dismutase (SOD), and endothelial nitric oxide synthase (eNOS) Enzyme Activity Assay Kits, and nitric oxide (NO), angiotensin-converting enzyme (ACE), vascular endothelial cell growth factor (VEGF), intercellular adhesion molecule-1 (ICAM-1), and nuclear factor-kappa B (NF-*κ*B) ELISA Kits were purchased from Xinzetianyou Biotechnology Co., Ltd. (Beijing, China). All chemicals and reagents were of analytical grade.

### 2.2. Antibodies

Rabbit monoclonal primary antibody against Nox4 was purchased from Santa Cruz Biotechnology, Inc. (Santa Cruz, CA). Rabbit polyclonal primary antibody against Akt was purchased from MengZhouShi Ruiying Biological Technology Co. Ltd. (Jiaozuo, Henan, China). Mouse monoclonal primary antibody against VEGF was purchased from Abcam (Shanghai, China). Rabbit monoclonal primary antibody against *β*-actin was purchased from Sigma Aldrich (Shanghai, China). Goat anti-mouse/rabbit IgG HRP-linked secondary antibodies were purchased from Cell Signaling Technology Inc. (Shanghai, China). Primary antibodies were used at 1 : 1000 dilutions. Secondary antibodies were used at 1 : 4000 dilutions.

### 2.3. Extraction of Anthocyanins from Blueberries

Extraction of anthocyanins from blueberries was performed according to the method used previously by Liu et al. [18]. The stored blueberry fruits were thawed at room temperature and beaten to a pulp. The blueberry pulp (250 g) was soaked in 1000 mL of methanol containing 1% HCl solution for 24 h by mixing every two hours to extract anthocyanins. The extract was collected and centrifuged at 5000 ×g for 15 min. After in vacuo evaporation of the solvent at 40°C, the residue was dissolved in 300 mL double distilled water and extracted with 1 : 1 (*v*/*v*) ethyl acetate three times to remove impurities, such as phenolic acids. The water phase containing anthocyanins was collected and concentrated in vacuo to obtain the crude anthocyanin extract. The extract was further purified with AB-8 macroporous resin (Sigma Aldrich, China). The extract was subjected to column chromatography on 1000 g AB-8 macroporous resin for 24 h absorption and then eluted with double distilled water to remove fructose and protein. Anthocyanin fraction was eluted with 80% ethanol containing 1% HCl solution, concentrated in vacuo, and then dried using an Eyela FDU-1200 freeze dryer (Tokyo Rikakikai, Japan) to get blueberry anthocyanin extract (BAE) powder (0.3 g). Blueberry anthocyanin extract was characterized by HPLC-DAD to determine the exact amount of each compound in the extract. Malvidin glycosides were the dominant anthocyanins in the blueberries, accounting for 47.9% of total anthocyanin content, in which Mv-3-glc and Mv-3-gal were 17.2% and 22.6%, respectively (unpublished data).

### 2.4. Endothelial Cell Culture and Treatment

HRCECs were cultured in normal glucose (5.5 mmol/L) DMEM containing 10% FBS and 1% streptomycin and penicillin at 37°C in a 5% CO_2_ humidified incubator. The third to fifth passage cells were used for all experiments at 80–90% confluence. HRCECs were quiesced in a reduced serum medium (not containing FBS) for 4 h before the experiment. Based on the preliminary experiments, a dose-response assay regarding viability showed that the extract and compounds were not toxic to the cells at a concentration of less than 10 *μ*g/mL. The cells were seeded in 6-well plates and pretreated with 10 *μ*g/mL of BAE, Mv, Mv-3-glc, or Mv-3-gal for 24 h, respectively. Then, the sample cells were stimulated with high glucose (final concentration 30 mmol/L) for another 24 and 48 h. Normal glucose (NG: 5.5 mmol/L) group without pretreating blueberry anthocyanins was used as the control. High glucose (HG: 30 mmol/L) group without pretreating blueberry anthocyanins was used as the oxidative model. The supernatants were collected for ELISA analysis. The cells were prepared for Western blotting.

### 2.5. Cell Viability Detection

The cell viability was determined by MTT method. The cells were cultured with high glucose (30 mmol/L) for 24 and 48 h with or without BAE, Mv, Mv-glc, or Mv-gal pretreatment for 24 h. Ten microliters of MTT (5 mg/mL) was added to each well and cultured for 4 h. After the removal of MTT solution, cell crystal was dissolved by adding 100 *μ*L DMSO (dimethylsulfoxide) and shaking 10 min slowly. The absorbance was measured at 550 nm on a Synergy H4 Multi-Mode Microplate Reader (BioTek Instruments, Inc., Winooski, VT, USA). The reader was controlled via Hyper Terminal Applet ELISA software. The cells were cultured only when normal glucose (5.5 mmol/L) was used as the control group. The blank group used the wells without cells. The cell viability was determined with the following formula: Cell viability(%) = (sample group OD value − blank group OD value)/(control group OD value − blank group OD value) × 100%.

### 2.6. Reactive Oxygen Species (ROS) Assay

Dichloro-dihydro-fluorescein diacetate (DCFH-DA) detection kit was used to assess the ROS level in HRCECs. Briefly, the cells were seeded in 96-well plates, treated with different samples to incubate for 24 h and cultured with high glucose (30 mmol/L) for 24 and 48 h. After washing cells with phosphate-buffered saline (PBS), 10 *μ*mol/L DCFH-DA was added to each well and reacted for 20 min at 37°C. The cells were collected after dissociation, and fluorescence was recorded by a Synergy H4 Multi-Mode Microplate Reader (BioTek Instruments, Inc., Winooski, VT, USA) with 488-P excitation and 525-P emission filters. The total fluorescence intensity of cells in each well was noted, and ROS generation was measured as fold of the control (each NG group, 24 and 48 h).

### 2.7. ELISA Analysis and Western Blotting

The levels of CAT, SOD, NO, eNOS, ACE, VEGF, ICAM-1, and NF-*κ*B were quantified from the supernatants using ELISA Kits or enzyme activity assay kits. The assay procedure was employed according to the kit protocol booklet instructions. The absorbance was measured at 450 nm on a Synergy H4 Multi-Mode Microplate Reader (BioTek Instruments, Inc. Winooski, VT, USA). The reader was controlled via Hyper Terminal Applet ELISA software. The total cell protein of the supernatant in each well was detected using BCA Protein Assay Kit.

Protein expression of Nox4, Akt, and VEGF was also analyzed by Western blotting performed on the HRCEC lysates. Data were normalized by reprobing the membrane with an antibody against *β*-actin, which was used as a loading control. Cell lysates from untreated cells were loaded on to every gel, and all data were expressed as fold of the corresponding control.

### 2.8. Statistical Analysis

All data presented are mean value ± standard deviation (SD) of three independent experiments. Figures were obtained using GraphPad Prism Version 5 (GraphPad Software, Inc., CA, USA). The data of each group used *t*-tests were to determine significant differences among different treatments (NG, HG, BAE, Mv, Mv-3-glc, and Mv-3-gal). Two-way analysis of variance (ANOVA) was used to analyze differences in culture time (24 and 48 h) and their interactions with treatment. The differences were considered significant at *P* value < 0.05.

## 3. Results

### 3.1. Effects of Blueberry Anthocyanins on Cell Viability

**Figure 1:**
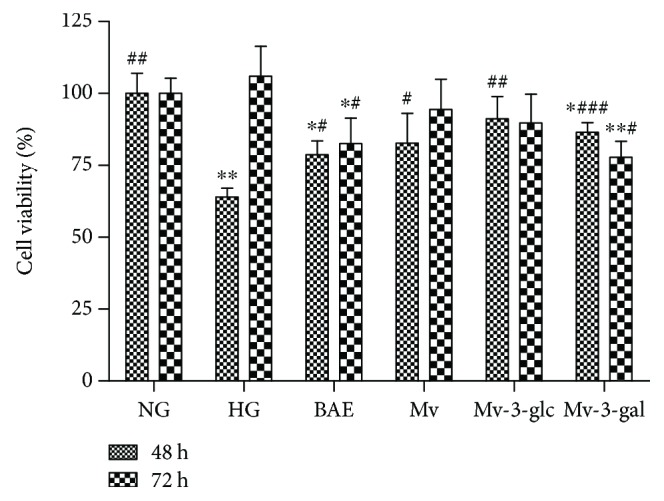
Effects of different treatment on cell viability in HRCECs exposed to high glucose for 24 and 48 h. ∗ and ∗∗ indicate *P* < 0.05 and *P* < 0.01, respectively, compared to each NG group; ^#^, ^##^, and ^###^ indicate *P* < 0.05, *P* < 0.01, and *P* < 0.001, respectively, compared to each HG group.

In this study, we observed that stimulation with high glucose concentration (30 mmol/L, HG: 64.03 ± 2.97%) for 24 h significantly decreased cell viability in comparison with normal glucose- (5.5 mmol/L, NG: 100 ± 7.04%) incubated cells (*P* < 0.01). MTT assay showed that 10 *μ*g/mL of BAE, Mv, Mv-3-glc, and Mv-3-gal all significantly increased cell viability after 24 h of high glucose incubation (*P* < 0.05) (Figure 1). The effect of blueberry anthocyanin extract (BAE: 78.69 ± 4.75%) was lower than those of malvidin and its glycosides, while the effects of malvidin glycosides were stronger than that of malvidin (Mv: 82.78 ± 10.27%). Malvidin-3-glucoside (Mv-3-glc: 91.16 ± 7.77%) was better than malvidin-3-galactoside (Mv-3-gal: 86.49 ± 3.32%). These indicated that high glucose inhibited cell growth, while BAE, malvidin, and its glycosides protected the cell by inhibiting a decrease in cell viability caused by HG. Interestingly, the same effects were not found in cells incubated with HG for a long time (significant difference between 24 and 48 h: *P* < 0.01). The cells continued to grow over time with more cell numbers at 48 h than at 24 h in each well. However, no significant difference was observed between NG (100 ± 5.28%) and HG (106.01 ± 10.41%), while anthocyanin pretreatments seemed to decrease a little cell viability (BAE: 82.55 ± 8.90%, Mv: 94.42 ± 10.49%, Mv-3-glc: 89.76 ± 10.01%, and Mv-3-gal: 77.84 ± 5.57%).

### 3.2. Effects of Blueberry Anthocyanins on ROS, CAT, SOD, and Nox4 Levels

**Figure 2:**
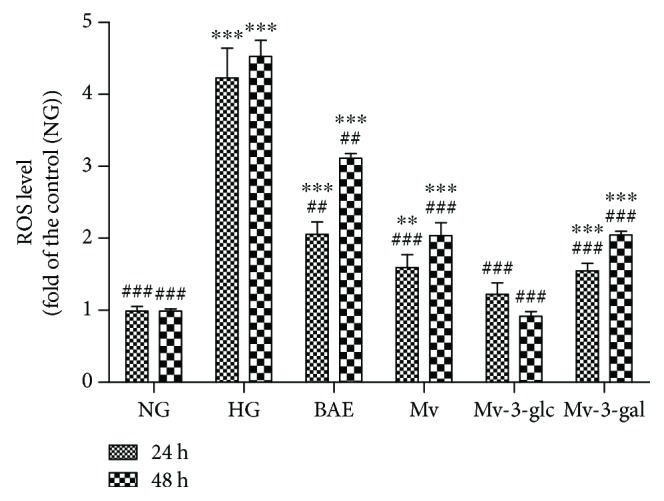
Effects of different treatment on ROS levels in HRCECs exposed to high glucose for 24 and 48 h. ∗∗ and ∗∗∗ indicate *P* < 0.01 and *P* < 0.001, respectively, compared to each NG group; ^##^ and ^###^ indicate *P* < 0.01 and *P* < 0.001, respectively, compared to each HG group.

As shown in Figure 2, high glucose concentration significantly enhanced the ROS levels of HRCECs at both short-term and a long-term (*P* < 0.001). ROS levels of the HG groups at 24 and 48 h were 4.23 ± 0.41- and 4.53 ± 0.23-folds of the control (NG groups), respectively. BAE, Mv, Mv-3-glc, and Mv-3-gal all significantly inhibited HG-induced increase of ROS in HRCECs (*P* < 0.01 for 24 h; *P* < 0.001 for 48 h). HRCECs exhibited higher ROS levels at 48 h than those at 24 h (*P* < 0.001) except pretreatment with Mv-3-glc. Similarly, malvidin-3-glucoside possessed the strongest antioxidant effect, with nearly the same ROS levels as the control. Moreover, the ROS level of cells treated with Mv-3-glc was only 0.93 ± 0.23-fold of the control when exposed to high glucose for 48 h. These data suggest that BAE, malvidin, and their glycosides significantly reduced the levels of ROS, showing an effective attenuation of high glucose-induced oxidative damage in HRCECs.

**Figure 3:**
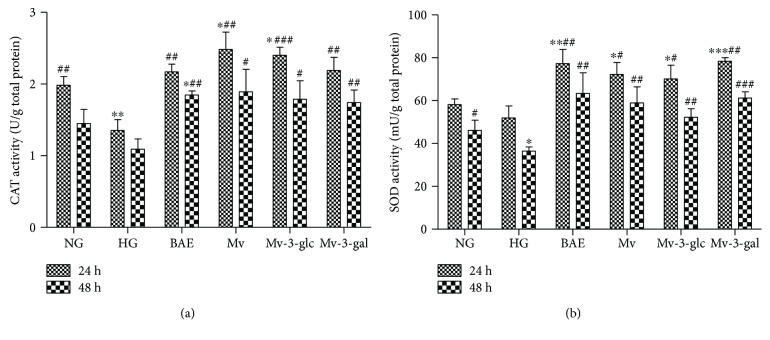
Effects of different treatment on CAT (a) and SOD activity (b) in HRCECs exposed to high glucose for 24 and 48 h. ^∗^, ^∗∗^, and ^∗∗∗^ indicate *P* < 0.05, *P* < 0.01, and *P* < 0.001, respectively, compared to each NG group; ^#^, ^##^, and ^###^ indicate *P* < 0.05, *P* < 0.01, and *P* < 0.001, respectively, compared to each HG group.

In this study, high glucose reduced the activity of the antioxidant enzymes CAT (*P* < 0.01 at 24 h) and SOD (*P* < 0.05 at 48 h) in HRCEC supernatants (Figure 3). BAE, Mv, Mv-3-glc, and Mv-3-gal all significantly enhanced CAT and SOD activity in HRCEC supernatants (*P* < 0.05). The CAT and SOD enzyme activity of cells pretreated with BAE, Mv, Mv-3-glc, and Mv-3-gal was even higher than that of the control (NG group). BAE and Mv-3-gal seemed to have more pronounced effects on SOD activity than Mv and Mv-3-glc. However, Mv and Mv-3-glc seemed to have more pronounced effects on CAT activity than BAE and Mv-3-gal. Both CAT and SOD enzyme activity decreased in a time-dependent manner (24 h versus 48 h: *P* < 0.01). Thus, blueberry anthocyanin malvidin and its glycosides could attenuate oxidative stress by upregulating the antioxidant enzymes activities in HRCECs.

**Figure 4:**
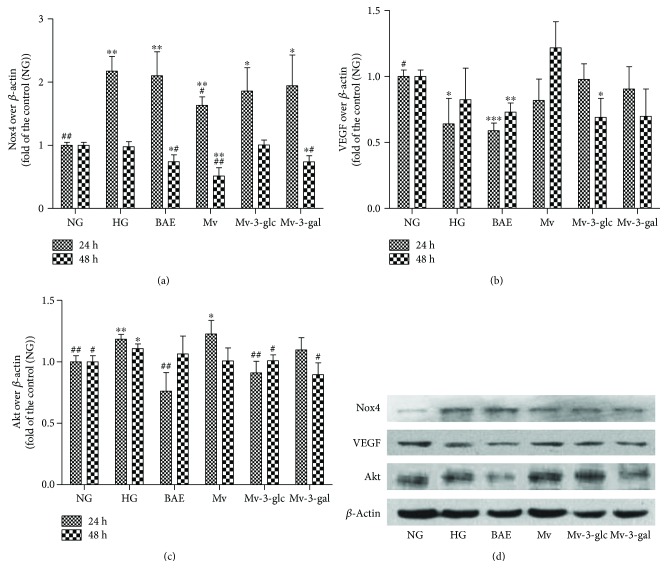
Effects of different treatment on Nox4 (a), VEGF (b), and Akt (c) protein expression in HRCECs exposed to high glucose for 24 and 48 h. (d) Representative Western blots of HRCECs exposed to high glucose for 24 h are shown. ^∗^, ^∗∗^, and ^∗∗∗^ indicate *P* < 0.05, *P* < 0.01, and *P* < 0.001, respectively, compared to each NG group; ^#^ and ^##^ indicate *P* < 0.05 and *P* < 0.01, respectively, compared to each HG group.

Figure 4(a) showed that high glucose significantly enhanced Nox4 protein expression in cells to 2.17-fold of the control after 24 h stimulation (*P* < 0.01), but it had no significant change for 48 h stimulation. Only Mv could greatly inhibit Nox4 expression induced by high glucose stimulation for 24 h with inhibition rate at 45.96% (*P* < 0.05). BAE, Mv, and Mv-3-gal all could lower Nox4 levels in cells with 48 h HG stimulation (*P* < 0.05 for BAE and Mv-3-gal; *P* < 0.01 for Mv). However, Mv-3-glc did not affect the Nox4 protein expression in cells at both 24 and 48 hours. Nox4 protein expression also decreased in a time-dependent manner (24 h versus 48 h: *P* < 0.01). The results indicated that Mv could reduce superoxide production in endothelial cells by downregulating Nox4. Mv-3-glc inhibited the most ROS formation but did not inhibit Nox4 expression, which should exist some other mechanism for Mv-3-glc on antioxidant effects in HRCECs.

### 3.3. Effects of Blueberry Anthocyanins on NO, eNOS, and ACE Levels

**Figure 5:**
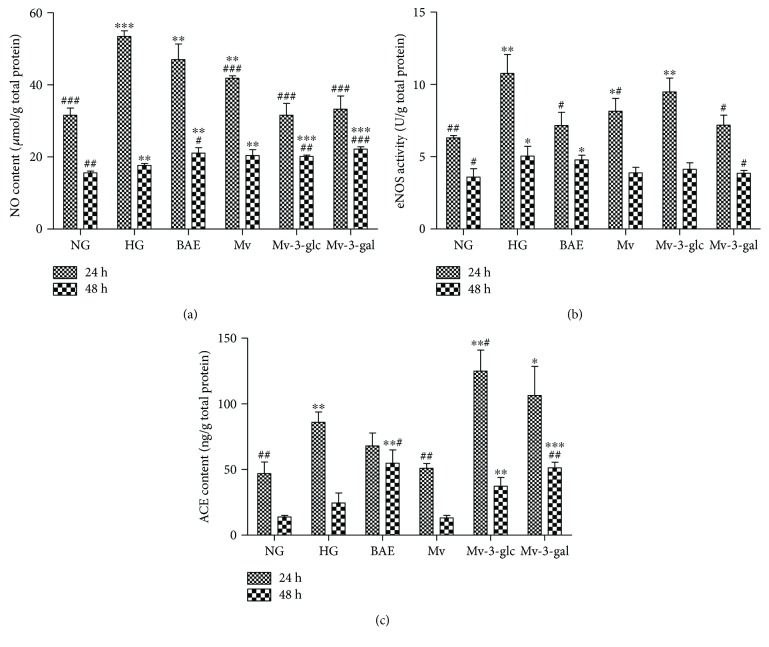
Effects of different treatment on NO contents (a), eNOS activity (b), and ACE contents (c) in HRCECs exposed to high glucose for 24 and 48 h. ^∗^, ^∗∗^, and ^∗∗∗^ indicate *P* < 0.05, *P* < 0.01, and *P* < 0.001, respectively, compared to each NG group; ^#^, ^##^, and ^###^ indicate *P* < 0.05, *P* < 0.01, and *P* < 0.001, respectively, compared to each HG group.

In this study, high glucose could significantly increase the NO level (*P* < 0.001 at 24 h; *P* < 0.01 at 48 h) and eNOS activity (*P* < 0.01 at 24 h; *P* < 0.05 at 48 h) in HRCEC supernatants (Figures 5(a) and 5(b)). BAE slightly inhibited the increase of NO at 24 h (*P* > 0.05) but not as much as Mv-3-glc and Mv-3-gal (*P* < 0.001). However, Mv-3-glc and Mv-3-gal seemed to induce more NO than the HG group for long time incubation (*P* < 0.01 for Mv-3-glc and *P* < 0.001 for Mv-3-gal at 48 h). HRCECs produced higher NO at 24 h than those at 48 h. Enzyme activity of eNOS in HRCECs pretreated with BAE, Mv, Mv-3-glc, and Mv-3-gal was higher than that of NG and lower than that of HG. However, the differences among them were not always significant. Like CAT and SOD, eNOS enzyme activity decreased in a time-dependent manner (24 h versus 48 h: *P* < 0.001). The change of ACE content in the supernatants showed that high glucose-induced ACE expression (*P* < 0.01 at 24 h). Malvidin downregulated the HG-induced ACE expression, while BAE, Mv-3-glc, and Mv-3-gal further upregulated ACE expression (Figure 5(c)). ACE contents also decreased in a time-dependent manner (24 h versus 48 h: *P* < 0.001).

### 3.4. Effects of Blueberry Anthocyanins on High Glucose-Induced VEGF and Akt Levels

**Figure 6:**
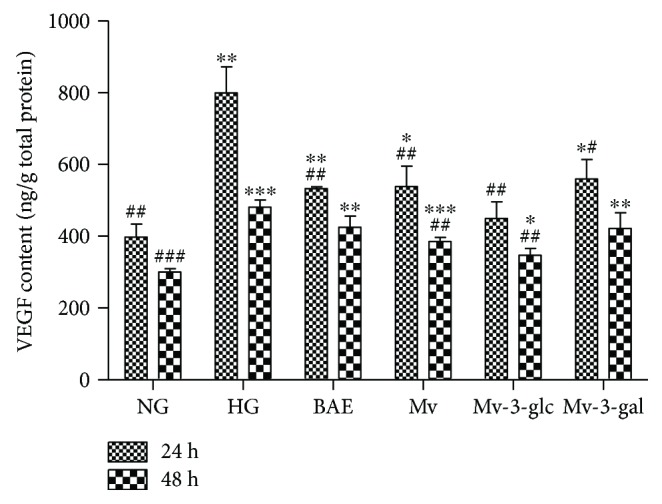
Effects of different treatment on VEGF contents in HRCECs exposed to high glucose for 24 and 48 h. ^∗^, ^∗∗^, and ^∗∗∗^ indicate *P* < 0.05, *P* < 0.01, and *P* < 0.001, respectively, compared to each NG group; ^#^, ^##^, and ^###^ indicate *P* < 0.05, *P* < 0.01, and *P* < 0.001, respectively, compared to each HG group.

Figure 6 shows that high glucose concentration could significantly upregulate the VEGF production in HRCEC supernatants (*P* < 0.01 at 24 h; *P* < 0.001 at 48 h). BAE, Mv, Mv-3-glc, and Mv-3-gal significantly inhibited HG-induced VEGF production at 24 h (*P* < 0.01 for BAE, Mv, and Mv-3-glc; *P* < 0.05 for Mv-3-gal). Mv and Mv-3-glc also exhibited inhibitory effects at 48 h (*P* < 0.01). However, BAE and Mv-3-gal did not show the VEGF inhibitory effect at 48 h. Like the contents of NO and ACE and the enzyme activity of CAT, SOD, and eNOS, VEGF contents were decreased with a prolonged time (24 h versus 48 h: *P* < 0.001). Mv-3-glc showed the strongest inhibitory effects against VEGF.

VEGF protein expression in cells was also determined by Western blotting (Figure 4(b)). Strangely, high glucose did not enhance VEGF protein expression in cells and even 24 h HG stimulation decreased VEGF level to 0.64-fold of the control (*P* < 0.05). BAE could greatly inhibit VEGF expression both at 24 h (0.59-fold of the control) and 48 h (0.73-fold) (*P* < 0.001 and *P* < 0.01 versus the control). Moreover, Mv-3-glc exhibited lower VEGF protein level (0.69-fold) than the control (*P* < 0.05). VEGF protein expression in cells at 24 and 48 h was different (*P* < 0.05). The VEGF levels at 48 h pretreated with BAE and Mv were higher than those at 24 h, which was consistent with the HG-stimulated group. However, the VEGF levels at 48 h pretreated with Mv-3-glc and Mv-3-gal were lower than those at 24 h, which indicated their capacity to downregulate the VEGF expression.

Moreover, high glucose could enhance the expression of Akt protein in cells to 1.18- (*P* < 0.01) and 1.11- (*P* < 0.05) folds of the control after 24 and 48 h stimulation, respectively. BAE, Mv-3-glc, and Mv-3-gal inhibited the increased Akt expression with HG stimulation for 24 h or 48 h. The Akt protein levels of BAE, Mv-3-glc, and Mv-3-gal at 24 h were 0.76-, 0.91-, and 1.09-folds of the control, respectively, and at 48 h, they were 1.06-, 1.01-, 0.89-folds of the control, respectively (Figure 4(c)). However, Mv did not affect the Akt protein expression in cells.

### 3.5. Effects of Blueberry Anthocyanins on High Glucose-Induced ICAM-1 and NF-*κ*B

**Figure 7:**
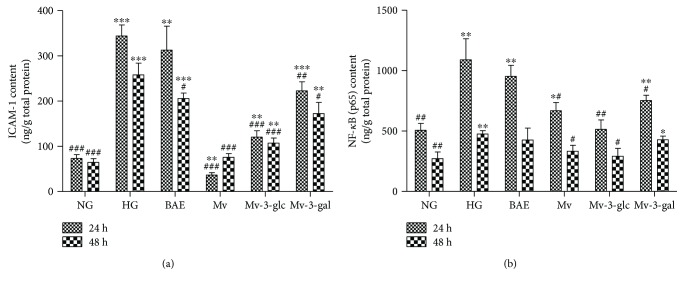
Effects of different treatment on ICAM (a) and NF-*κ*B (p65) contents (b) in HRCECs exposed to high glucose for 24 and 48 h. ^∗^, ^∗∗^, and ^∗∗∗^ indicate *P* < 0.05, *P* < 0.01, and *P* < 0.001, respectively, compared to each NG group; ^#^, ^##^, and ^###^ indicate *P* < 0.05, *P* < 0.01, and *P* < 0.001, respectively, compared to each HG group.

In this study, high glucose concentrations could significantly upregulate the ICAM-1 production in HRCEC supernatants (*P* < 0.001 both at 24 and 48 h). As shown in Figure 7(a), malvidin and Mv-3-glc greatly inhibited HG-induced ICAM-1 levels (*P* < 0.001 both at 24 and 48 h), particularly Mv exhibited a lower ICAM-1 content than NG at 24 h (*P* < 0.01). Mv-3-gal was also observed to have an ICAM-1 inhibitory effect compared to the HG group (*P* < 0.01 at 24 h and *P* < 0.05 at 48 h). However, BAE had no effects on ICAM-1 levels in HRCECs at 24 h. ELISA assay of NF-*κ*B (p65) contents showed similar results. However, the effects were not always significant (Figure 7(b)). Moreover, Mv and Mv-3-glc exhibited strongest inhibitory effects at both 24 and 48 h (*P* < 0.05). The ICAM-1 contents and NF-*κ*B (p65) levels of 48 h were lower than those of 24 h (*P* < 0.001) except Mv, which had a lower ICAM-1 content at 24 h than that at 48 h (*P* < 0.01).

## 4. Discussion

The chronic hyperglycemia-induced cell death in the endothelial cells of retinal vessels is well established [2]. Leal et al. found that high glucose, but not mannitol, decreased cell viability of rat retinal endothelial cells exposed to 30 mM glucose (high glucose) for 7 days (long-term exposure) [20]. Fan et al. also observed that stimulation with 30 mM glucose for 48 h and 72 h significantly decreased cell viability in comparison with 5.5 mM glucose-treated rat retinal capillary endothelial cells [1]. However, Wang et al. reported that high glucose-induced human retinal microvascular endothelial cell proliferation and enhanced the cell viability [2]. In the present study, cell viability of human retinal capillary endothelial cells was decreased by high glucose only when stimulated for 24 h but not for 48 h. Maybe the effect of cell proliferation counteracted the effect of cell death at 48 h, so there was no significant change in cell viability. Moreover, blueberry anthocyanin extract and its major constituents (malvidin glycosides) could have a particular protective effect on HRCECs, all enhancing cell viability. Their possible functional mechanisms include attenuation of oxidative damage, alteration of retinal enzyme activity, and inhibition of inflammation [6]. As a whole extract, BAE exhibited a reductionist activity, since crude extract also had some other noneffective components. This confirmed that anthocyanins, particularly Mv-3-gal and Mv-3-glc, were effective components in blueberry extracts.

The upregulated reactive oxygen species (ROS) generation inferred the potent stimuli for the oxidative stress [21]. In this study, BAE, Mv, Mv-3-glc, and Mv-3-gal attenuated high glucose-induced oxidative stress in HRECE by reducing ROS levels. The activity of nicotinamide adenine dinucleotide phosphate (NADPH) oxidases has been identified as an important source of ROS in vascular endothelial cells [22]. Nox4 is the major catalytic component of an endothelial NADPH oxidase. The self-perpetuating cycle between oxidative stress and inflammation contributes to the upregulation of NADPH oxidase, which increases ROS production [23]. The ROS can react with nitric oxide (NO) to form peroxynitrate leading to a reduction in NO bioavailability and subsequently impaired NO-dependent vasodilation [15]. However, Schröder et al. speculated that endogenous Nox4 protects the vasculature against inflammatory stress because loss of Nox4 results in the reduction of endothelial nitric oxide synthase (eNOS) and heme oxygenase-1 (HO-1) expression, as well as NO production, which comprise an important antioxidant defense against endothelial oxidative damage [23]. This might explain why Nox4 did not change greatly with pretreatment with blueberry anthocyanin malvidin glycosides in this study.

The local production of NO mediates the function of endothelium-dependent vasodilation, which is synthesized from *L*-arginine by the enzyme eNOS [24]. Conversely, angiotensin-converting enzyme (ACE) is responsible for vasoconstriction by converting angiotensin I (AngI) into AngII (a potent vasoconstrictor) and inactivating the vasodilator bradykinin [25]. Vasodilation and vasoconstriction of blood vessels together affect blood pressure. In several pathological conditions including diabetes, endothelium-dependent vasodilation is reduced because of a decreased release of NO [15, 26]. Extracts from various plants full of anthocyanins can induce endothelium-dependent vasodilation probably by releasing NO or enhancing NO bioactivity [27]. For example, blackcurrant prevented eye probably by increasing blood supply based on the endothelium vasodilation [28]. Strangely, the effects on NO production were not always consistent with the effects on eNOS activity in this study. The level of NO could not be completely determined by eNOS because endothelial NOS is only one of the three isoforms of nitric oxide synthase [29]. Other isoforms, such as neuronal NOS (nNOS) and inducible NOS (iNOS), should also be effective. Retinal capillary endothelial cells can express iNOS when stimulated by inflammatory mediators, such as IL-1*β* [21]. Moreover, nitrosative/oxidative stress might be related to NO levels. NOS can also catalyze superoxide anion production, depending on substrate and cofactor availability [29]. Anthocyanins exhibited high NO levels, which contributed to the effective antioxidant capacity and the vasodilatory effect. Our previous study found that malvidin and its glycosides could inhibit TNF-*α*-induced ACE expression and activity in the human umbilical vein endothelial cells [30]. However, blueberry anthocyanins did not downregulate ACE expression and were not detected in HRCECs in the present study. It seemed to be contradictory that BAE and malvidin glycosides possessed simultaneous vasodilatory and vasoconstrictive effects at the same time. Clozel et al. also reported that the endothelin ETB receptor could mediate both vasodilation and vasoconstriction [31]. Further study on the mechanism of malvidin glycosides in the endothelium should be conducted in the future.

Under retinopathy state, the activity of NOS is spontaneously regulated to improve the formation of NO fighting for inflammation [29]. Moreover, the stimulus-secretion coupling of high glucose-induced the synthesis and the release of NO could interact with the vascular endothelial growth factor (VEGF) [32]. VEGF stimulates vasculogenesis and angiogenesis, which gives rise to the proliferative DR [33]. In the diabetic microangiopathic condition, microvascular permeability and the number of leucocytes sticking to the vascular endothelium are increased [6]. In the present study, high glucose stimulated VEGF secretion, and blueberry anthocyanin malvidin glycosides downregulated the VEGF expression.

Akt, the serine/threonine kinase, is a central node in cell signaling downstream responses, including cell survival, growth, proliferation, angiogenesis, glucose uptake, metabolism, and migration [34]. In endothelial cells, the PI3K-Akt pathway is robustly activated by VEGF, and Akt activates eNOS [35]. The release of NO produced by activated eNOS can stimulate vasodilation, vascular remodeling, and angiogenesis [36]. During diabetic retinopathy, Akt activation is aberrant. In the present study, blueberry anthocyanin malvidin glycosides inhibited Akt expression, therefore inhibiting eNOS activity and changing the NO level.

Nuclear factor-kappa B (NF-*κ*B), an important protein complex that controls the transcription of DNA and cytokine production, is present in many cell types and participates in cell apoptosis and neovascularization [37]. On activation of the NF-*κ*B pathway, the p65 protein interacts with the promoters to induce and maintain the state of inflammation [38]. It has been shown that the expression of NF-*κ*B p65 in the retina can regulate the expression of intercellular adhesion molecule-1 (ICAM-1), which is the primary adhesion molecule responsible for inflammation in the pathogenesis of diabetic retinopathy [39]. Previous studies confirmed that Mv-3-glc could increase NO bioavailability as well as inhibit peroxynitrite-induced NF-*κ*B activation [40]. Anthocyanin and phenolic acid metabolites were found to attenuate visible light-induced retinal degeneration in vivo via NF-*κ*B suppression [41]. In the present study, blueberry anthocyanin malvidin glycosides also exhibited anti-inflammatory effects by inhibiting NF-*κ*B in HRCECs.

## 5. Conclusions

In the present study, blueberry anthocyanin extract, as well as its major constituent malvidin and its glycosides, could protect human retinal capillary endothelial cells against high glucose-induced injury. BAE, Mv, Mv-3-glc, and Mv-3-gal promoted cell growth of HRCECs with higher cell viability than the high glucose-stimulated group. They had great antioxidant effects by decreasing ROS levels and increasing enzyme activity of CAT and SOD in HRCECs. Downregulation of Nox4 expression might be an antioxidant mechanism. Moreover, the upregulation of NO levels might be another antioxidant mechanism for blueberry anthocyanins, which contributed to vasodilatory effects. However, malvidin glycosides still possessed vasoconstrictory effects by increasing ACE contents in some case. Blueberry anthocyanins and malvidin glycosides changed VEGF levels in HRCECs and influenced the Akt pathway to some extent. Moreover, Mv and Mv-3-glc significantly inhibited HG-induced extracellular ICAM-1 and NF-*κ*B (p65). BAE, Mv, Mv-3-glc, and Mv-3-gal protected cells in a time-dependent manner with the difference between 24 and 48 h HG stimulation. Incubation for long times may weaken the protective effects, which can be attributed to the irreparable oxidative damage caused by the prolonged stimulation of high glucose. The results indicated that blueberries, as an excellent resource of anthocyanins, could improve human retinal capillary endothelial function and, thereby, might have the potential to prevent the progression of diabetic retinopathy.

## Supplementary Materials

Supplementary Materials Figure 1. Chromatographic separation and UV detection (*λ* = 520 nm) of anthocyanins in blueberry anthocyanin extract. The following 14 peaks were identified: 1, delphindin-3-galactoside; 2, delphindin-3-glucoside; 3, cyaniding-3-galactoside; 4, petunidin-3-galactoside; 5, cyaniding-3-glucoside; 6, cyaniding-3-arabinoside; 7, petunidin-3-glucoside; 8, peonidin-3-galactoside; 9, petunidin-3-arabinoside; 10, peonidin-3-glucosidea; 11, malvidin-3-galactoside; 12, malvidin-3-glucoside; 13, malvidin-3-arabinose; and 14, acylated anthocyanin.
