# Supplementary material for: Antioxidant and Anti-Inflammatory Effects of Blueberry Anthocyanins on High Glucose-Induced Human Retinal Capillary Endothelial Cells
Source: Oxid Med Cell Longev. 2018 Feb 22;2018:1862462. doi: 10.1155/2018/1862462 (PMC5842687; doi:10.1155/2018/1862462)
Supplement: Supplementary Materials — Figure 1. Chromatographic separation and UV detection (λ = 520 nm) of anthocyanins in blueberry anthocyanin extract. The following 14 peaks were identified: 1, delphindin-3-galactoside; 2, delphindin-3-glucoside; 3, cyaniding-3-galactoside; 4, petunidin-3-galactoside; 5, cyaniding-3-glucoside; 6, cyaniding-3-arabinoside; 7, petunidin-3-glucoside; 8, peonidin-3-galactoside; 9, petunidin-3-arabinoside; 10, peonidin-3-glucosidea; 11, malvidin-3-galactoside; 12, malvidin-3-glucoside; 13, malvidin-3-arabinose; and 14, acylated anthocyanin. [file 1862462.f1.doc]

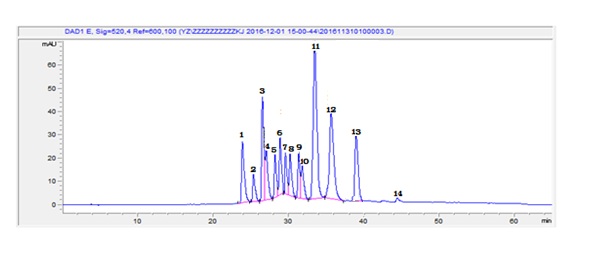


Figure.1. Chromatographic separation and UV detection (λ = 520 nm) of anthocyanins in blueberry anthocyanin exact. The following 14 peaks were identified: 1, delphindin-3-galactoside; 2, delphindin-3-glucoside; 3, cyaniding-3-galactoside; 4, petunidin-3-galactoside; 5, cyaniding-3-glucoside; 6, cyaniding-3-arabinoside; 7, petunidin-3-glucoside; 8, peonidin-3-galactoside; 9, petunidin-3-arabinoside; 10, peonidin-3-glucosidea; 11, malvidin-3-galactoside; 12, malvidin-3-glucoside; 13, malvidin-3-arabinose; 14, acylated anthocyanin.
